# One Size Fits All? The Validity of a Composite Poverty Index Across Urban and Rural Households in South Africa

**DOI:** 10.1007/s11205-016-1540-x

**Published:** 2016-12-27

**Authors:** Janina Isabel Steinert, Lucie Dale Cluver, G. J. Melendez-Torres, Sebastian Vollmer

**Affiliations:** 1 Department of Social Policy and Intervention, University of Oxford, Barnett House, 32 Wellington Square, Oxford, OX1 2ER UK; 2 Department of Psychiatry and Mental Health, University of Cape Town, Cape Town, South Africa; 3 Warwick Medical School, University of Warwick, Coventry, CV4 7AL UK; 4 Chair of Development Economics, University of Göttingen, Platz der Göttinger Sieben 3, 37073 Göttingen, Germany; 5 Department of Global Health and Population, Harvard T.H. Chan School of Public Health, Boston, MA USA

**Keywords:** Asset indices, Multidimensional poverty, Poverty indices, Structural equation modelling, Validity

## Abstract

Composite indices have been prominently used in poverty research. However, validity of these indices remains subject to debate. This paper examines the validity of a common type of composite poverty indices using data from a cross-sectional survey of 2477 households in urban and rural KwaZulu-Natal, South Africa. Multiple-group comparisons in structural equation modelling were employed for testing differences in the measurement model across urban and rural groups. The analysis revealed substantial variations between urban and rural respondents both in the conceptualisation of poverty as well as in the weights and importance assigned to individual poverty indicators. The validity of a ‘one size fits all’ measurement model can therefore not be confirmed. In consequence, it becomes virtually impossible to determine a household’s poverty level relative to the full sample. Findings from our analysis have important practical implications in nuancing how we can sensitively use composite poverty indices to identify poor people.

## Introduction

Composite indices have been critical to the understanding of poverty in both research and policy. One of the most widely used examples is the Human Development Index (HDI) that has been developed by the United Nations Development Program. The HDI seeks to measure household wealth and wellbeing by aggregating a range of welfare-related aspects into an overall index and then ranking countries according to their performance on the three dimensions of life expectancy, education, and income per capita. In a similar vein, asset indices have been widely used for the measurement of household welfare (e.g. Filmer and Scott 2012; Carter and Barrett 2006; Sahn and Stifel 2000, 2003; Filmer and Pritchett 2001; Moser and Felton 2007). An asset index is defined so that household wealth increases (numerically) with the possession of more durables/assets and a higher standard of living, for instance through access to electricity and running water. The *Demographic and Health Survey* collects extensive data on quality of housing and household assets in the majority of low- and middle-income countries and has thus further promoted reliance on these indices. Another example of a composite measurement is the Multidimensional Poverty Index (MPI) that was developed by the Oxford Poverty and Human Development Initiative (OPHI) and has gained international standing over the past decade. The MPI assesses individual deprivation profiles based on a set of ten indicators including aspects of nutrition, mortality, schooling, and decent standards of living. For each of these ten indicators, individuals are classified as ‘deprived’ or ‘non-deprived’ based on pre-defined cut-offs. Countries are then ranked according to the headcount ratio of people who experience multiple deprivations (Alkire et al. 2016a, b; Alkire and Santos 2014; OPHI 2013; Alkire and Foster 2011; Ferreira and Lugo 2013). From 2010 onwards, the MPI has been integrated in the Human Development Report that is released annually by the United Nations Development Program (UNDP 2010).

Despite widespread use, the validity of composite poverty indices remains contested. Sahn and Stifel (2003) and Filmer and Pritchett (2001) demonstrate the robustness of such indices, particularly when it comes to identifying the poorest groups of society (Klasen 2000). However, others have questioned their adequacy when used over time and across space (Harttgen et al. 2013; Saisana et al. 2005). A commonly cited concern is the use of ‘one-size-fits-all’ measurement approaches across urban and rural locations (Chakraborty et al. 2016; Douidich et al. 2015; Stifel and Christiaensen 2007; Vyas and Kumaranayake 2006). For instance, OPHI has now moved towards disaggregating MPI rankings for urban and rural populations (Alkire et al. 2016b) and Rutstein (2008) advocates for the application of urban- and rural-specific DHS Wealth Index. Yet, despite these cautionary tales, composite poverty indicators are still commonly used without taking sub-national (as well as cross-national) heterogeneity into account, both in high-level policy reports (World Bank 2015; UNDP 2015; OECD 2015) as well as academic outlets (Hruschka et al. 2015; Smits and Steendijk 2014; Batana 2013; Michelson et al. 2013; Booysen et al. 2008). The present analysis draws upon these two strands of literature by generating further empirical evidence to demonstrate that the rural–urban divide *does* matter for aggregated poverty indices. We draw on data across urban and rural locations of the KwaZulu-Natal province in South Africa. Significant disparities in urban and rural locations of South Africa mirror former spatial policies of the Apartheid regime (Daniels et al. 2013; Klasen 2000). Previous studies have identified large gaps in levels of income and deprivation (Klasen 2000; Sahn and Stifel 2000), employment (Turok 2012), or receipt of governmental grants (Daniels et al. 2013) between urban and rural households. Income migration to peri-urban and urban centres has further shaped these spatial disparities in South Africa (Posel 2004). KwaZulu-Natal’s population is a largely homogenous ethnic group (Zulu) and the same political party has governed the former homeland since 1994. The province therefore appears an ideal location for isolating differences between urban and rural areas from some important potential confounders.

## Data and Methods

### Data

The analysis uses data from a larger study^1^ in KwaZulu-Natal, South Africa. Data were collected between 2009 and 2010, sampling both deep rural (Manguzi/kwaNgwanase) and peri-urban (Lamontville/Umlazi townships) communities of the province. The sampling methodology followed the South African census model of stratified systematic random sampling. Stratification was done per census enumeration area or per designated tribal area in rural locations that were identified through Geographical Information System. Respondents were then selected through door-to-door household sampling. A 10–17-year-old child/adolescent was randomly selected in each household and asked to identify his/her primary caregiver, defined as the person living with and responsible for the day-to-day care of the child. While the child survey put focus on a range of psychosocial outcomes; household characteristics and socioeconomic information were collected via caregiver reports. The present analysis therefore draws on cross-sectional data from the household survey with 2477 caregivers. Face-to-face interviews were conducted by local research assistants in isiZulu with both adolescents and their primary caregivers. Participation was voluntary and informed consent was sought from all respondents. The ethical protocol was approved by the University of Oxford, the University of KwaZulu-Natal, and the provincial Department of Health and Education (see also Cluver et al. 2013). Sociodemographic information was collected using items from the South African Census (2001) and the South Africa General Household Survey. Indicators for housing quality and ownership of assets were based on the Demographic and Health Surveys (DHS). All measures are specified in detail in “Appendix”.

### Construction of a Composite Poverty Index

For the purpose of this paper, we operationalize poverty in the form of a composite index that aggregates a range of individual indicators into a scalar score. The core idea hereby is to move beyond material aspects and capture the broader dimensions of wellbeing such as health, education, or social capital (Sen 1993). Based on a review of the literature on poverty measurements, Table 1 provides a comprehensive overview of the different indicators conventionally used for the construction of poverty indices. Although there are some variations in the number and kind of indicators, most approaches have covered three main dimensions reflective of individuals’ experienced deprivation and poverty. A first dimension is *housing quality* which may relate to hygiene and general health outcomes (Rutstein and Johnson 2004; Klasen 2000). Secondly, *ownership of assets* can have several welfare implications: Household assets can serve as a security buffer against economic shocks (Zimmerman and Carter 2003), livestock ownership can secure nutritional needs (Cohen and Saisana 2014), and a means of transport can translate into improved medical care and higher participation in social life. The last dimension is *human capital* which includes nutritional health, schooling, educational attainment, and employment; all of which have a range of positive externalities such as health-relevant knowledge, potential for income generation, as well as providing a source of self-respect and fulfilment (Sen 1993).

**Table 1:** Poverty indicators: overview

Indicator	Prior application	Relation to poverty
Safe drinking water	Qi and Wu (2014), Harttgen et al. (2013), Échevin (2013), DHS (2012), Alkire and Foster (2011), UNDP (2010), Sahn and Stifel (2003), Battiston et al. (2013), Booysen et al. (2008), Klasen (2000), Montgomery et al. (2000)	Access to clean water can improve hygiene and general health. Household access to a source of clean water can free up productive time from fetching water
Toilet facilities (e.g. flush toilet or pit latrine)	Qi and Wu (2014), Harttgen et al. (2013), Échevin (2013), DHS (2012), Alkire and Foster (2011), UNDP (2010), Battiston et al. (2013), Wright (2008), Booysen et al. (2008), Moser and Felton (2007), Sahn and Stifel (2003), Filmer and Pritchett (2001)	Good sanitation can improve hygiene and general health
Cooking fuel	Qi and Wu (2014), DHS (2012), Alkire and Foster (2011), UNDP (2010), Sahn and Stifel (2003), Filmer and Pritchett (2001), Klasen (2000)	Use of unprocessed solids leads to indoor air pollution, poor respiratory health and is correlated with high accident rates
Heating fuel	Qi and Wu (2014)	Indoor air pollution and high accident rates
Lighting	Moser and Felton (2007), Filmer and Pritchett (2001)	General housing quality
Number of rooms/over-crowding	Échevin (2013), DHS (2012), Wright (2008), Filmer and Pritchett (2001)	Several person per sleeping room is related to increased transmission of respiratory illnesses
Electricity	Qi and Wu (2014), DHS (2012), Alkire and Foster (2011), UNDP (2010), Wright (2008), Montgomery et al. (2000)	General housing quality
Floor	Qi and Wu (2014), Harttgen et al. (2013), Échevin (2013), DHS (2012), Alkire and Foster (2011), UNDP (2010), Booysen et al. (2008), Moser and Felton (2007), Sahn and Stifel (2003), Montgomery et al. (2000)	General housing quality
Wall	Harttgen et al. (2013), DHS (2012), Battiston et al. (2013), Moser and Felton (2007)	General housing quality
Dwelling	DHS (2012), Wright (2008), de Kruijk and Rutten (2007), Filmer and Pritchett (2001)	General housing quality
Bicycle/motorcycle	Qi and Wu (2014), Harttgen et al. (2013), Ferreira and Lugo (2013), Échevin (2013), DHS (2012), UNDP (2010), Booysen et al. (2008), Wright (2008), Moser and Felton (2007), Sahn and Stifel (2003), Filmer and Pritchett (2001), Klasen (2000), Montgomery et al. (2000)	Basic transportation is linked to better access to healthcare and community/social life
Car	Échevin (2013), DHS (2012), Alkire and Foster (2011), UNDP (2010), Wright (2008), Moser and Felton (2007), Klasen (2000), Montgomery et al. (2000)	Transport affects the ability to participate in labor market and society
Refrigerator	Qi and Wu (2014), Harttgen et al. (2013), Ferreira and Lugo (2013), DHS (2012), Alkire and Foster (2011), Échevin (2013), Booysen et al. (2008), Wright (2008), Moser and Felton (2007), Sahn and Stifel (2003), Filmer and Pritchett (2001), Klasen (2000), Montgomery et al. (2000)	Household wealth accumulated in durables/assets
Washing machine	Qi and Wu (2014), DHS (2012), Wright (2008), Moser and Felton (2007)	Household wealth accumulated in durables/assets
TV	Qi and Wu (2014), Harttgen et al. (2013), Ferreira and Lugo (2013), Échevin (2013), DHS (2012), Alkire and Foster (2011),UNDP (2010), Wright (2008), Booysen et al. (2008), Moser and Felton (2007), Sahn and Stifel (2003), Filmer and Pritchett (2001), Klasen (2000), Montgomery et al. (2000)	Household wealth accumulated in durables/assets
Computer	Qi and Wu (2014), Moser and Felton (2007)	Household wealth accumulated in durables/assets
Telephone	Qi and Wu (2014), Ferreira and Lugo (2013), DHS (2012), Alkire and Foster (2011), UNDP (2010), Wright (2008), Klasen (2000)	Household wealth accumulated in durables/assets
Radio	Harttgen et al. (2013), Échevin (2013), DHS (2012), UNDP (2010), Wright (2008), Booysen et al. (2008), Moser and Felton (2007), Sahn and Stifel (2003), Filmer and Pritchett (2001), Montgomery et al. (2000)	Household wealth accumulated in durables/assets
Livestock	DHS (2012), UNDP (2010), Bishai et al. (2005)	Household wealth accumulated in durables/assets, may secure basic nutritional needs
Education/schooling	Qi and Wu (2014), Échevin (2013), DHS (2012), Sahn and Stifel (2003), UNDP (2010), Battiston et al. (2013), Moser and Felton (2007), Montgomery et al. (2000)	Human capital, increased competitiveness on labour market, increased health knowledge
Employment	DHS (2012), Wright (2008), de Kruijk and Rutten (2007), Klasen (2000), Montgomery et al. (2000)	Income source and basis for self-respect and fulfillment
Food/hunger	Qi and Wu (2014), Alkire and Foster (2011), de Kruijk and Rutten (2007)	

The table is based on a comprehensive literature search. The electronic databases MEDLINE, social sciences citation index (SSCI), applied social sciences index and abstracts (ASSIA), global health, and Proquest dissertations and theses were searched (last update: July 2014). Additional relevant studies were identified through back referencing. Relevant grey literature was retrieved by screening the databases of UNAIDS, WHO, and the World Bank

There are four types of aggregating individual indicators into a poverty scale. A first way assigns *equal weights* to each individual indicator such as in the HDI (for a critique, see Ravallion 2011; Filmer and Pritchett 2001). Alternatively, weights can be based on *expert opinions* and ethical deliberations of policy makers (OPHI 2013; for a critique, see Cohen and Saisana 2014). Thirdly, weights can be defined through *participatory approaches* and assigned according to priority patterns of the population of interest (Wright and Noble 2007; Noble et al. 2004; Barnes and Wright 2012). Lastly, scholars have used *statistical procedures* such as factor analysis or principal component analysis and assign weights based on correlation structures between a range of individual poverty indicators (Shaffer 2013; Sahn and Stifel 2003; Filmer and Pritchett 2001).

This paper aligns with the statistical approach for aggregating individual indicators from Table 1 into a composite poverty index.^2^ It is hereby assumed that each of these indicators reflects an underlying and unobserved variable that denotes household poverty. The index is designed to maximise discrimination between poorer and wealthier households. This is achieved by assigning higher weights to those poverty items that display more variation across households. In other words, assuming that every household owns a telephone, the item would be given a weight of zero as it would not adequately distinguish between worse and better off households. In the same vein, if no household were to own a car, the weight would again turn zero. Following this, each individual indicator is first assigned a specific and distinct weight before indicators are then summed up. The procedure yields a continuous scale in which higher scale scores denote a higher level of household poverty. Factor loadings for household assets and quality of housing will therefore be negative considering that possession or access *decreases* severity of poverty. The above process can be represented in the following equation:

$${\text{P}}_{\text{i}} = {\text{y}}_{1} {\text{p}}_{{{\text{i}}1}} + \cdots + {\text{y}}_{\text{k}} {\text{p}}_{\text{ik}} +\updelta{\text{i}}$$

where P_i_ denotes the poverty scale score, p_ik_ the respective poverty indicators, y_k_ the weights (factor loadings) for each indicators and δ_i_ a stochastic error term (Sahn and Stifel 2003).

### Analyses

Statistical analysis was done in three steps. First, exploratory factor analysis (EFA) was used in order to explore whether data was loading on a single or multiple factors and to eliminate irrelevant individual indicators. Screeplots and Eigenvalues were inspected for selecting factors (see Field 2009). An Eigenvalue indicates the amount of variance that a factor explains in a set of observed variables. In a screeplot variance is plotted against the number of principal components and serves to visually assess which factors explain most of the variability in the variance in the data. Consequently, items are assessed according to their factor loading. A factor loading reflects the strength of association of an individual variable with the underlying factor. Following Costello and Osborne (2005), we use a factor loading of 0.3 and above as orientation. Internal consistency was first assessed for items in the full sample and then for the urban and rural samples separately.

Second, following McKenzie (2003), kernel-density estimates for the poverty index were examined whereby a roughly normal distribution would suggest that ‘clustering’ or ‘clumping’ is unlikely That is, asset ownership and achievements should be independent from whether a respondent belongs to a certain geographical area or sub-population (i.e. cluster) Tests for normality were conducted using the Shapiro–Wilk-test.

A third step introduced structural equation modelling (SEM). Goodness of fit and internal consistency of the proposed measurement model was examined. Multiple-group comparisons in SEM were utilized for testing potential differences in the proposed measurement model across groups (Steenkamp and Baumgartner 1998). In this process, measurement invariance of the proposed poverty framework was assessed across groups, in this case urban and rural households. Invariance of the poverty measurement model is gradually increased and the fit of each subsequent model assessed (Steenkamp and Baumgartner 1998). At first, *configural invariance* is introduced, which requires the item structure to be maintained (that is, the same set of indicators is aggregated), but allows different loadings on each item (Acock 2013). Configural invariance can thereby assess whether poverty has the same meaning across groups or whether some items reflect social status in one society while they are less important in another society. Secondly, *metric invariance* is tested by constraining factor loadings to be invariant across groups. If factor loadings vary by sub-group, indicator weights are different for each group. In consequence, scale scores are based on different mathematical procedures; poverty rankings can therefore not be meaningfully compared across the full sample (Steenkamp and Baumgartner 1998; Meredith 1993). Thirdly, *scalar invariance* is tested, whereby intercepts of the underlying items are constrained to be equal across groups. If scalar invariance cannot be confirmed, it is possible that the design of the composite measure is biased against one of the groups and that observed poverty values differ systematically from latent poverty values. Model fit was assessed for each of the above three steps (Steenkamp and Baumgartner 1998; Meredith 1993). For this purpose, we used the Chi square goodness-of-fit test, the comparative fit index (CFI), the root mean error of approximation (RMSEA), and the standardized root mean square residual (SRMR). Conventional cut-offs indicating a *good* model fit require the values of CFI to be over 0.95 (and lower than 0.05 for RMSEA and SRMR. A CFI of 0.90 and RMSEA/SRMR of 0.08 may still be considered as a *reasonable* fit (see Schreiber et al. 2006; Hu and Bentler 1999). Poor fit would suggest that a single composite poverty index may be less valid and reliable for measuring household poverty across rural and urban sites within South Africa.

## Results

### Descriptive Statistics

52% of households were located in urban and 48% in rural locations. Urban and rural interviewees were largely similar in terms of gender (female: 90% in rural, 87% in urban households), age (average of 45.2 years in rural, 43.2 years in urban households), and ethnic origin (94% Zulu in urban, 98% in rural households) of interviewed caregivers. Likewise, the structural composition of households (in terms of breadwinning and caregiving) was fairly similar: the average age of all household members was 23.5 years in urban and 18.0 years in rural households and the percentage of household members >60 years was 2.5% in urban and 4.1% in rural households. Table 2 displays all individual poverty indicators, stratified by urban and rural residency. Ownership of most assets is significantly higher in urban locations. Similarly, urban respondents appear significantly less food-insecure and have higher levels of education. In addition, unemployment is less prevalent in urban households, but could conceivably reflect labour migration to urban centres (Posel et al. 2006). In contrast, possession of livestock is higher in rural areas which may well be indicative of agricultural productivity, self-subsistence, income generation, as well as high property crime and limited space in urban areas (Batana 2013; Booysen et al. 2008). Further, while sanitation and building material appear more sophisticated in urban households, overcrowding—defined as the number of people sleeping in one room—is significantly higher in urban households. Importantly for intergenerational poverty, the average number of children who dropped out or are currently not attending school is significantly higher in urban areas.

**Table 2:** Household poverty in urban and rural Kwa-Zulu Natal

	Urban	Rural
Continuous variables		
Number of children not attending school	M 0.94	M 0.76
Ratio: children attending to children not attending school	M 1.56	M 2.12
Overcrowding: ratio household members per room	M 5.08	M 3.50
Categorical variables		
Hunger		
Never	65.2%	54.4%
Seldom	17.6%	19.6%
Sometimes	15.0%	25.0%
Often	2.2%	1.0%
Education		
No schooling	2.3%	36.7%
Primary school	15.7%	37.4%
Secondary school	54.2%	17.7%
Matric	26.2%	7.5%
University	1.6%	0.7%
Employment		
Permanent	17.9%	7.3%
Temporary	15.2%	13.5%
Unemployed	67.0%	79.2%
Binary variables		
Meal with meat	90.9%	65.4%
Computer	10.4%	2.4%
TV	90.0%	38.1%
Radio	88.1%	62.6%
Refrigerator	84.9%	29.9%
Drinking source	87.6%	26.9%
Safe water	98.6%	82.0%
Washing machine	9.5%	0.3%
Electricity	94.5%	9.9%
Cooking	98.9%	6.6%
Heating	61.8%	1.8%
Lighting	93.6%	6.9%
Toilet	84.1%	2.1%
Floor	97.0%	93.5%
Wall	71.6%	67.5%
Dwelling	73.4%	63.3%
Phone	96.1%	93.2%
Car	11.5%	12.3%
Bicycle	5.5%	5.9%
Motorcycle	0.9%	0.2%
Cattle or sheep	0.6%	11.6%
Donkey or horse	0.5%	0.8%
N	1279	1197

Means displayed for continuous variables. For categorical variables, cells display the distribution of each category. For binary variables, each cell displays the percentage of households who indicate possession of item

### Exploratory Factor Analysis

**Fig. 1:**
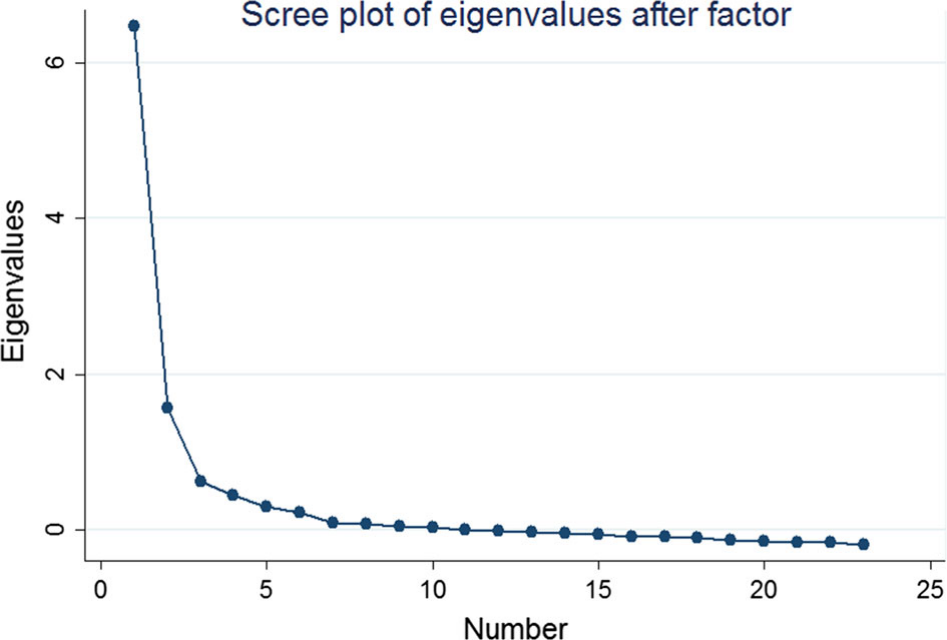
Exploratory factor analysis: scree plot

EFA initially included all items listed in Table 1. Inspection of screeplots and Eigenvalues suggested a single-factor solution (see Fig. 1). The first factor had an Eigenvalue of 6.47 and appeared to be the only strong factor, explaining 75.6% of the variance in the 23 poverty items.

Table 3 displays factor loadings for each individual item. Looking at the results of the EFA for the full sample, most items had loadings >0.3. After removing items with particularly low factor loadings (floor quality, overcrowding, possession of phone, livestock, car/bike, washing machine) the poverty scale had high internal reliability, with Cronbach’s α = 0.86.

**Table 3:** Summary of single-factor exploratory factor analysis

Item	Full sample (n = 2353)	Rural sample (n = 1212)	Urban sample (n = 1140)
Hunger	0.25	0.36	0.21
Refrigerator	−0.74	−0.59	−0.64
Phone	−**0**.**15**	−**0**.**15**	−**0**.**18**
Computer	−0.26	−0.20	−0.24
Transport	−**0**.**18**	−0.44	−0.28
Meat	−0.42	−0.36	−0.25
TV	−0.71	−0.55	−0.68
Radio	−0.45	−0.36	−0.44
Drinking source	−0.67	−0.31	−0.58
Safe water	−0.27	−**0**.**01**	−**0**.**13**
Washing machine	−0.28	−**0**.**11**	−0.22
Electricity	−0.90	−0.58	−0.72
Cooking	−0.87	−0.54	−**0**.**16**
Heating	−0.67	−0.39	−0.30
Lighting	−0.80	−0.61	−0.68
Toilet	−0.91	−0.24	−0.41
Floor	−**0**.**18**	−0.25	−0.20
Wall	−0.32	−0.54	−0.68
Dwelling	−0.39	−0.55	−0.66
Overcrowding	**0**.**04**	0.30	**0**.**02**
Livestock	**0**.**14**	−**0**.**14**	−**0**.**07**
Education	−0.61	−0.36	−0.21
Employment	−0.23	−0.25	−**0**.**10**
Eigenvalues	*6*.*47*	*3*.*56*	*4*.*01*
% of variance	*75.6*	*67.5*	*60.8*
α^a^	*0.86*	*0.70*	*0.72*

Information on children’s schooling was not available for all sampled households. The variable was thus excluded from EFA so as to keep the size of the sample

^a^Excluding items with factor loadings low factor loadings (highlighted in bold and italics)

We then examined factor loadings for the urban and rural sub-samples separately. This showed that factor loadings varied considerably between the sub-samples. While some indicators appear important and are thus assigned a higher weight in one setting, they become less relevant in the other (see Vyas and Kumaranayake 2006). For instance, ownership of a vehicle appears to be indicative of which rural households are better off than others, whereas it does not show comparable importance in urban settings, likely reflecting better access to public transport, closer services and therefore lower need for private transport. Likewise, household overcrowding and possession of livestock do not reliably demarcate poverty status in urban settings. In contrast, possession of a washing machine shows low factor loadings for rural households, likely because only a negligible part of rural households own a washing machine (0.3%).

### Distribution of Poverty

**Fig. 2:**
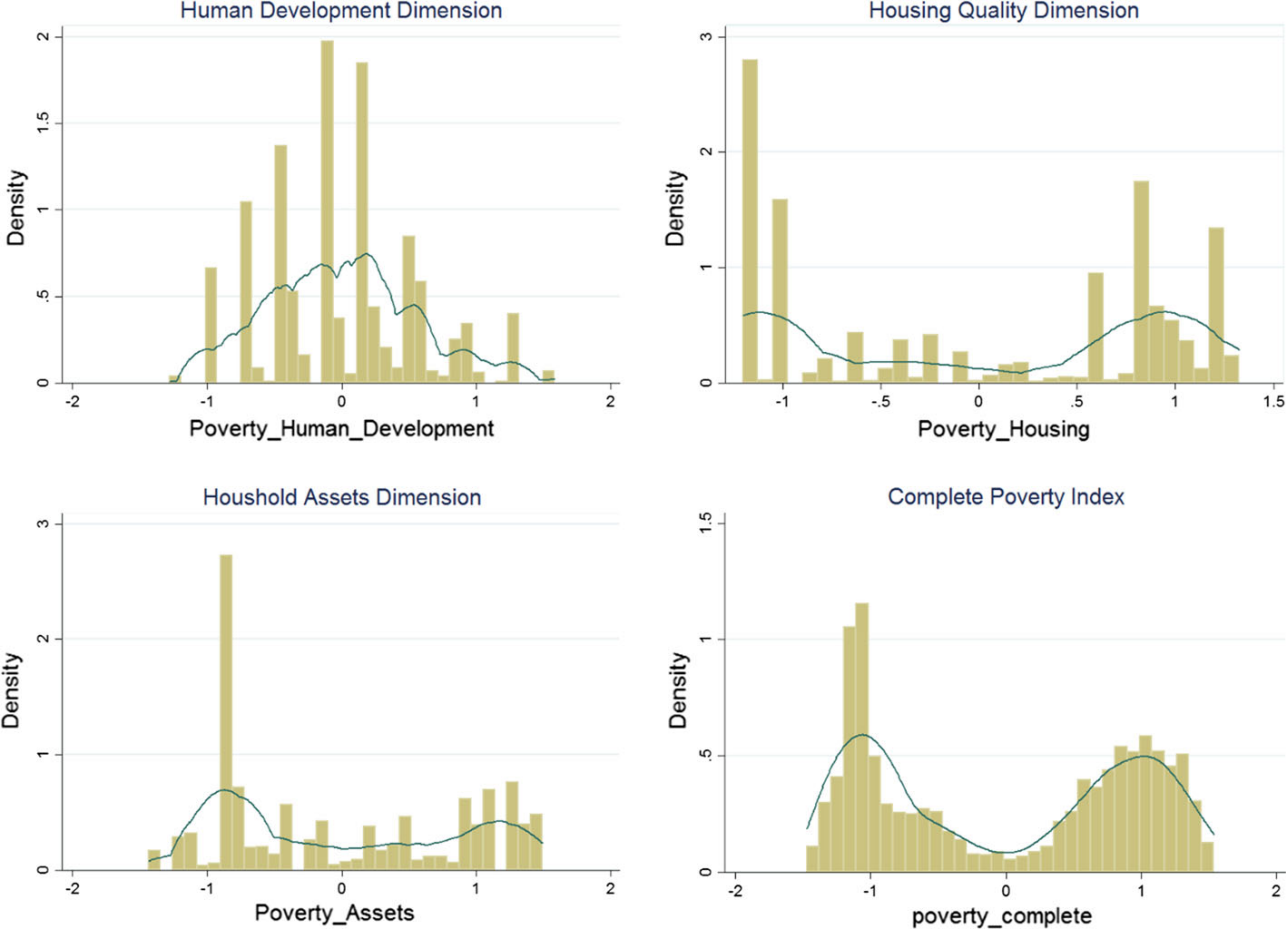
Histograms and kernel densities for the distribution of poverty indicators

Figure 2 displays histograms and kernel-density estimates for the distribution of different composite poverty indicators across urban and rural location. Three sub-indices were created to reflect three dimensions: (1) human capital/human development (including education, schooling, employment, and hunger), (2) housing quality, and (3) asset ownership. The last graph in Fig. 2 examines the complete composite poverty index. Following McKenzie (2003), the non-normal distribution of values on the separate dimensions and composite index suggest the presence of ‘clumping’. After displaying histograms for urban and rural subgroups separately, it becomes apparent that the two peaks in each distribution coincide with the two subgroups. That is, respondents appear to be clumped together in urban and rural groups and patterns of asset ownership and achievements are distinct from each other. Utilizing the Shapiro–Wilk-test, we rejected the null hypothesis that any of the above indices is normally distributed (*p* < 0.001). Further, we employ a rigorous statistical procedure for examining multimodality in the distribution of the complete composite poverty index. Utilizing Silverman’s (1981) non-parametric test, we test whether the above kernel density distribution has two (or more) modes against the null hypothesis of a single mode.^3^ We use 500 bootstrap replications for estimating the critical bandwidth of the distribution. Results of the Silverman test reject unimodality with a confidence level of *p* < 0.01. We cannot reject the null hypothesis of two modes in favour of three modes (*p* = 0.12), suggesting that the distribution has indeed two peaks that reflect the urban and rural samples.

### Structural Equation Model: Multiple Group Comparison

When fitting the above poverty index in a structural equation model, the four items of: access to a safe water source, possession of a phone, possession of livestock, and possession of a washing machine were removed as showing low factor loadings for both rural and urban groups in EFA. Introducing the structural equation model, goodness of fit of the original model proved low. To improve model fit, modification indices were inspected to inform possible changes to the model. Error terms were correlated based on substantial modification index values and conceptual logic (see Schreiber et al. 2006). This included correlated errors for the item pairs of wall and dwelling type, floor and dwelling type, hunger and meat, electricity and lighting, and education and employment. This considerably improved the fit of the measurement model (see Table 4) with CFI of 0.90.

**Table 4:** Measurement model of the poverty index

	Standardized	Unstandardized
Measurement model		
Hunger	0.19***	1 (fixed)
Meat	−0.38***	−0.99***
Overcrowding	0.04**	1.96**
Computer	−0.22***	−0.33***
Transport	−0.11***	−0.24***
TV	−0.67***	−2.00***
Radio	−0.41***	−1.09***
Refrigerator	−0.70***	−2.13***
Drinking source	−0.69***	−2.10***
Electricity	−0.90***	−2.80***
Cooking	−0.91***	−2.84***
Heating	−0.69***	−2.00***
Lighting	−0.92***	−2.85***
Toilet	−0.82***	−2.50***
Floor	−0.14***	−0.19***
Wall	−0.20***	−0.57***
Dwelling	−0.27***	−0.77***
Education	−0.60***	−3.78***
Employment	−0.21***	−0.90***
Covariances		
Error.meat with error.hunger	−0.24***	−0.08***
Error.electricity with error.lighting	0.64***	0.03***
Error.floor with error.dwelling	0.05***	0.01***
Error.wall with error.dwelling	0.72***	0.14***
Error.education with error.employment	0.13***	0.07*
Goodness of fit		
χ^2^	2455.108***	
CFI	0.83	
RMSEA	0.08	
SRMR	0.90	

Turning to multiple group comparisons, the average level of poverty was found to be substantially higher in the rural sample (M 0.14, SD 0.07) as compared to the urban sample (M −0.15, SD 0.06) (note: higher scale scores representing higher poverty). Following this, the procedural steps proposed by Steenkamp and Baumgartner (1998) were introduced. Table 5 displays the model fit for all three types of invariance tests and reveals that *configural invariance*—the model with the fewest constraints—had a CFI of 0.83 and thus did not display acceptable fit. Model fit became again weaker with every additional constraint on measurement invariance (CFI 0.79 for metric invariance and CFI 0.0 for scalar invariance). This finding points to a difference in the meaning of poverty between rural and urban households and to variations in the importance of specific poverty items across locations. Hence, validity and adequacy of the suggested poverty indicator could not be confirmed across the two populations.

**Table 5:** Goodness-of-fit indices for original and modified model

Model	χ^2^	CFI	RMSEA	SRMR
Configural invariance	2198.289 *p* < 0.001	0.83	0.07	0.06
Metric invariance	2641.092 *p* < 0.001	0.79	0.08	0.09
Scalar invariance	12,112.490 *p* < 0.001	0.00	0.17	0.30

Table 6 displays multiple-group comparisons for the model with the most acceptable fit. In this model, constraints are put on the number and kind of indicators used, but loadings are not required to be equal. The results strongly suggest that each sub-population assigns different importance to respective items. Generally speaking, asset ownership, sanitation, and energy appear to be of higher relevance in urban households, whereas employment and education turn out to be more emphasised in rural households. Overcrowding is found to be inadequate for measuring household poverty status in urban areas, as the item loading is non-significant for this sub-group.

**Table 6:** Multiple-group SEM for urban and rural sub-populations

	Urban (N = 1144)	Rural (N = 1212)
Measurement model		
Hunger	0.21***	0.34***
Meat	−0.27***	−0.35***
Overcrowding	0.01	0.28***
Computer	−0.20***	−0.20***
Transport	−0.24***	−0.46***
TV	−0.76***	−0.57***
Radio	−0.49***	−0.38***
Refrigerator	−0.71***	−0.62***
Drinking source	−1.11***	−0.32***
Electricity	−0.65***	−0.51***
Cooking	−0.08***	−0.54***
Heating	−0.84***	−0.40***
Lighting	−0.60***	−0.54***
Toilet	−0.33***	−0.23***
Floor	−0.17***	−0.23***
Wall	−0.56***	−0.43***
Dwelling	−0.53***	−0.45***
Education	−0.18***	−0.37***
Employment	−0.09***	−0.27***
Covariances		
Error.meat with error.hunger	−0.17***	−0.22***
Error.electricity with error.lighting	0.82***	0.63***
Error.floor with error.dwelling	0.04	0.05*
Error.wall with error.dwelling	0.68***	0.64***
Error.education with error.employment	0.19***	0.07*
Goodness of fit		
χ^2^	2198.29***	
CFI	0.83	
RMSEA	0.07	
SRMR	0.06	

* *p* < 0.05; ** *p* < 0.01; *** *p* < 0.001

## Discussion and Concluding Remarks

We set out to test the validity of a composite poverty index across urban and rural locations within an ethnically homogenous population. The analysis revealed substantial variations in the meaning and conceptualisation of poverty among urban and rural respondents. Factor loadings for individual poverty indicators were found to differ significantly between populations. A considerable number of poverty items such as household overcrowding, transportation, or employment could distinguish adequately between poorer and wealthier households in one area, but were found to have little relevance to socioeconomic status in the other area. In addition, the analysis found indication of ‘clumping’ effects in the distribution of observed poverty levels—pointing to different patterns of asset ownership and housing quality in urban and rural populations. Equivalence in the measurement model could not be confirmed across groups. Poverty rankings along the constructed poverty scale would thus be subject to measurement error.

The measurement approach we employed in this paper was similar to composite poverty measures commonly used in literature on household poverty and development. However, as revealed by this analysis, the poverty index did not show cross-geographical validity, even in an ethnically homogenous population. In other words, it appears that poverty manifests differently and is perceived differently in urban and rural communities within one province in South Africa. The value that each population assigns to a certain indicator defines the magnitude of its weight for statistical aggregation. Yet, if weights vary in urban and rural populations, scale scores are derived from different mathematical procedures. Comparability becomes invalid. This finding is crucial as previous rankings and comparisons of household poverty levels across South Africa (or even across KwaZulu-Natal) could thus be less reliable than thought.

According to the composite index, household poverty and deprivation appeared more pronounced in rural areas. This is in line with a range of prior studies (Batana 2013; Bérenger et al. 2013; Rutstein and Johnson 2004). Tendencies were somewhat similar in descriptive statistics of this analysis, but a number of essential indicators such as overcrowding and educational attainment portrayed rural households on average as ‘better off’. Therefore, while the finding of higher rural deprivation may indeed have some validity, these findings suggest that the conceptualisation of conventionally used poverty indices and the selection of individual indicators may be biased against rural households (Booysen et al. 2007, 2008). A range of items such as electricity, sewerage, and access to piped water reflect available infrastructure and public service provision rather than reliably measuring a range of differences in poverty levels (Harttgen et al. 2013). Moreover, other indicators such as land ownership or agricultural assets that are presumably more strongly valued in rural areas, are usually absent in conventional composite poverty measurements (Batana 2013; Vyas and Kumaranayake 2006). That is, poverty levels in rural families may indeed be higher, but the design of indices may also be prone to overestimating these differences.

This study has a number of limitations. Whilst an analysis like the one that we conducted here can test the validity of a composite poverty index within the context of South Africa, it would be interesting also to examine its performance across countries and over time. As Harttgen et al. (2013) describe, certain assets can become more accessible and prevalent over time (e.g. TVs and phones) and might thus become less adequate for classifying wealthier and poorer households. A ‘standard size’ poverty index would be subject to measurement bias as assigned weights would differ between one time point and the other (Vyas and Kumaranayake 2006).

Some more limitations are noteworthy that are inherent to all composite poverty measurements. A fist limitation lies in the binary nature of poverty indicators. That is, the aggregated index captures ownership of a certain asset, but not necessarily their quality, functionality, and possible depreciation over time (Harttgen et al. 2013; Falkingham and Namazie 2002). More importantly, most poverty indicators were measured at a household rather than individual level. Hence, there was no information on potential intra-household inequalities such as in education or nutrition. Specifically, there might be significant differences in resource distributions between female and male household members that could point to important gender gaps in a society (Harttgen et al. 2013), but that the present analysis could not detect. Further, we have tested validity in a very specific population and cannot claim generalizability of our findings.

This analysis hopes to contribute to the ongoing debate, as we strive towards the most effective means of both measurement and reduction of poverty in the developing world. Although great strides have been made in developing composite policy indices, further refinement may be required in order to identify whether these indices can serve as an adequate measurement tool for identifying the poorest and most deprived households, both within and across countries.

## Appendix: Poverty Indicators and Codings

**Table:**

Indicator	Coding	Measurement level
Possession of a bicycle, car, motorcycle => collapsed into one variable transport	Yes = 1 No = 0	Household
Possession of a refrigerator	Yes = 1 No = 0	Household
Possession of a telephone	Yes = 1 No = 0	Household
Possession of a TV	Yes = 1 No = 0	Household
Possession of a radio	Yes = 1 No = 0	Household
Possession of a computer	Yes = 1 No = 0	Household
Possession of sheep/cattle, donkey/horse => collapsed into one variable animals	Yes = 1 No = 0	Household
Meal with meat once a week	Yes = 1 No = 0	Individual (primary caregiver)
Hunger, i.e. people in household who are hungry	Ordinal variable Never = 1 Seldom = 2 Sometimes = 3 Often = 4	Household/Individual
Electricity in the house	Yes = 1 No = 0	Household
Main source of drinking water	Original categories 1. Piped water (tap) in dwelling 2. Piped water (tap) in site/yard 3. Bottled water 4. Public tap 5. Water carrier/tanker 6. Borehole/well 7. Dam/river/stream/spring 8. Rain-water tank 9. Neighbour’s tap 10. Other => Collapsed into a binary variable: access to piped water (1./2.) = 1 remaining categories = 0 Category “other” recoded manually (see Sahn and Stifel 2003, p. 469)	Household
Is the water from that source safe?	Yes = 1 No = 0	Household
Toilet type	Original categories 1. Flush toilet (own) 2. Flush toilet (shared) 3. Bucket latrine 4. Pit latrine 5. No facility/bush/field 6. Other => Collapsed into a binary variable: flush toilet = 1, remaining categories = 0 Category “other” recoded manually (see Sahn and Stifel 2003, p. 469; Filmer and Pritchett 2001, p. 117)	Household
Material of the floor	Original categories 1. Earth/sand/dung 2. Bare wood planks 3. Cement 4. Vinyl 5. Carpet 6. Ceramic tiles 7. Parquet or polished wood 8. Other => Collapsed into a binary variable: “smart” floor (no dirt,sand, dung or wood) = 1, remaining categories = 0 Category “other” recoded manually (see Booysen et al. 2008)	Household
Material of the wall	Original categories 1. Plastic/cardboard 2. Mud 3. Mud and cement 4. Corrugated iron/zink 5. Prefab 6. Bare brick 7. Cement block 8. Plaster/finished 9. Wooden planks 10. Other => Collapsed into a binary variable: cement/plastered/prefab wall = 1, remaining categories = 0 Category “other” recoded manually	Household
Dwelling type	Original categories 1. Dwelling/house or brick structure on a separate stand/yard 2. Town/cluster/semi-detached house 3. Dwelling/house/flat/room in backyard 4. Informal dwelling/shack in backyard 5. Informal dwelling/shack not in backyard 6. Room/flatlet on a property or a larger dwelling 7. Caravan/tent, other 8. Other => Collapsed into a binary variable: formal housing (brick structure, town/cluster/semi-detached house, house on someone else’s property, and apartment rentals) = 1, informal housing (shacks, caravans or tents, and traditional huts made of mud, stone, or wattle) = 0 Category “other” recoded manually	Household
Main source of energy for heating	Original categories 1. Electricity from mains 2. Electricity from generator 3. Gas 4. Paraffin 5. Wood 6. Coal 7. Candles 8. Animal dung 9. Solar energy 10. Other => Collapsed into a binary variable: electricity/gas = 1, remaining categories = 0 category “other” recoded manually (see Qi and Wu 2014, p. 94)	Household
Main source of energy for cooking	Original categories 1. Electricity from mains 2. Electricity from generator 3. Gas 4. Paraffin 5. Wood 6. Coal 7. Candles 8. Animal dung 9. Solar energy 10. Other => Collapsed into a binary variable: electricity/gas = 1, remaining categories = 0 Category “other” recoded manually (see Qi and Wu 2014, p. 94; Cohen and Saisana 2014, p. 36)	Household
Main source of energy for lighting	Original categories 1. Electricity from mains 2. Electricity from generator 3. Gas 4. Paraffin 5. Wood 6. Coal 7. Candles 8. Animal dung 9. Solar energy 10. Other => Collapsed into a binary variable: electricity/gas = 1, remaining categories = 0 category “other” recoded manually (see Qi and Wu 2014, p. 94)	Household
Schooling children	Three different variables Number of children in school Number of children not in school Ratio: number of children in school/number of children not in school per household	Household/Individual (each non-adult household member)
Education of primary caregiver	Original categories 1. No schooling 2. Grade R/0 3. Grade 1/sub A 4. Grade 3/standard 1 5. Grade 4/standard 2 6. Grade 5/standard 3 7. Grade 6/standard 4 8. Grade 7/standard 5 9. Grade 8/standard 6 10. Grade 9/standard 7 11. Grade 10/standard 8 12. Grade 11/standard 9 13. Certificate with less than grade 12/standard 10 14. Diploma with less than grade 12/standard 10 15. Grade 12/standard 10 16. Certificate with grade 12/standard 10 17. Diploma with grade 12/standard 10 18. NTC I/NTC II/NTC III 19. Bachelors degree 20. Honours 21. Higher degree masters, doctorate 22. Other => Collapsed into ordinal variable None = 1 Primary = 2 Secondary = 3 Matric = 4 University = 5	Individual (primary caregiver)
Employment	Ordinal variable No job = 0 Temporary employment = 1 Permanent employment = 2	Individual (primary caregiver)

## Footnotes

1 Young Carers Project South Africa: http://www.youngcarers.org.za/young-carers/.

2 While we refer to our composite measure as ‘poverty index’ throughout this paper, the index is designed to include a range of concepts including standard of living, quality of live, and wellbeing.

3 For a similar approach, see: Vollmer et al. (2013a, b, c).
